# Supplementary material for: Humans are more prosocial in poor foraging environments
Source: Nat Commun. 2026 Feb 9;17:483. doi: 10.1038/s41467-025-66880-9 (PMC12887026; doi:10.1038/s41467-025-66880-9)
Supplement: Supplementary file 1 — Supplementary Information [file 41467_2025_66880_MOESM1_ESM.pdf]

Supplementary Methods

Statistical analysis

Generalised linear mixed-effects models (GLMM) were used to predict participants' choices (accept or reject offer) in the Prosocial Ecology Task and the fitted computational parameters. Our main analyses included environment (poor vs. rich), recipient (self vs. other), and expected value (reward magnitude*probability) as well as their interactions as fixed effects. Continuous variables were centred within subjects and categorical variables were recoded using effect coding. Random effects included participant-level random intercepts, and random slopes that were determined by first fitting the maximal model and then comparing against reduced models to trim away random effects that did not improve the fit of the model in terms of Akaike Information Criterion (AIC; *1*). This was done to reduce Type I error while maintaining power, and improve model convergence (*1*, *2*). Force data in study 3 were normalised as the proportion of their maximum force and calculated using the area under the curve for the 3-second window in which they exerted force. The R and MATLAB code used for data analysis, visualisation, and model fitting are available at: https://doi.org/10.17605/osf.io/dmfhq.

Model comparison

In addition to using exceedance probability to compare models, as described in the main manuscript, we also compared the computational models using the integrated Bayesian Information Criterion (BICint), which tends to favour smaller models over complex ones. We found that the model with the lowest summed BICint had distinct opportunity costs for each recipient and environment, but only distinct value sensitivity parameters for each recipient (self vs. other). This model however had an exceedance probability close to 0 (the winning model had an exceedance probability of 0.96) and explained less variance than our winning model (R^2 = 0.82; winning model R^2 = 0.83). See **Figure S3** for the BICint scores for all models.

Supplementary Results

Study 2

Examining the effect of environment, we found that participants were less likely to act in the rich compared to the poor environment (poor vs. rich: OR = 0.29 [0.22, 0.37], z = 9.59, p < 0.001), and that this difference in environments widened at higher expected reward values (environment × expected value: OR = 0.17 [0.13, 0.22], z = 14.28, p < 0.001). Participants

also demonstrated that they were often willing to interrupt their behaviour to help others (M = 43.66%, SD = 22.77%, t-test comparing against 0%: $t_{(218)} = 28.37$, $p < 0.001$, $d = 1.92$ [1.73, 2.14]), despite having the option to continue watching the movie. However, as in study 1, participants showed a self-bias where they chose to act to help others less than opportunities for themselves (self vs. other: OR = 0.10 [0.07, 0.13], $z = 13.52$, $p < 0.001$). This difference self-bias also increased at higher expected values (recipient × expected value: OR = 0.37 [0.29, 0.46], $z = 8.45$, $p < 0.001$).

When we examined reward magnitude and probability as separate predictors, we again found that participants were more likely to choose to act at higher reward magnitudes (OR = 2.31 [2.06, 2.61], $z = 13.88$, $p < 0.001$) and at higher probabilities (OR = 7.89 [6.81, 9.16], $z = 27.44$, $p < 0.001$). This model (see **Table S9** for full results) revealed a significant 4-way interaction between reward magnitude, reward probability, environment, and recipient (OR = 0.74 [0.58, 0.96], $z = 2.31$, $p = 0.021$), analogous to the findings described above where reward magnitude and probability were computed together as expected value.

Participants in study 2 were also highly successful when they performed the effort task for both self and other. On average they succeeded in 99.6% of the effort task trials, which further supports the notion that the effort task did not meaningfully increase the risk of choosing to act. Examining participants' effort thresholds before and after the main task showed again that thresholds were higher at the end of the study ($t_{(217)} = 9.32$, $p < 0.001$, $d = 0.63$ [0.52, 0.75]), again suggesting that participants were not fatigued by the effort task. We also asked participants how much they enjoyed watching the movie and whether they had seen it previously. The majority of participants had not seen the movie before (175 *no* vs. 44 *yes*, $\chi^2_{(1)} = 78.36$, $p < 0.001$) and overall enjoyment was high (M (SD) = 6.89 (2.05), 0 = *did not enjoy at all,* 9 = *very much enjoyed*; t-test against neutral rating: $t_{(218)} = 17.27$, $p < 0.001$, $d = 1.17$ [0.94, 1.45]).

Comparing the number of credits earned we replicated the significant interaction between environment and recipient showing that, compared to themselves, participants earned relatively more credits for others when the environment was poor than when it was rich (recipient × environment: $b = –43.09$ [–51.00, –35.16], $z = 10.69$, $p < 0.001$). That is, although participants overall earned more credits for themselves than for others (self vs. other: $b = –36.61$ [–42.60, –30.62], $z = 12.03$, $p < 0.001$), and earned more credits in the rich blocks compared to the poor blocks (poor vs. rich: $b = 141.36$ [134.44, 148.27], $z = 40.25$, $p < 0.001$), the difference between credits earned for self and for other was smaller in the poor environment as compared to the rich one (**Figure S1**). Together, the findings from study 2

replicate our finding of robust ecological effects on prosocial decisions that are stronger than for the same decisions that benefit oneself.

Study 3

We also found that participants were again less likely to act to help the other person relative to themselves (self vs. other: OR = 0.20 [0.13, 0.31], z = 7.72, p < 0.001) and that this difference increased at higher expected values (recipient × expected value: OR = 0.32 [0.23, 0.43], z = 7.32, p < 0.001). There was also a significant effect of environment, replicating our findings from the studies described above. Overall, participants chose to act to benefit the other person 39.20% (*SD* = 16.57%) of the time (*t*-test comparing against 0%: $t_{(53)}$ = 17.39, p < 0.001, d = 2.37 [1.94, 3.07]). Participants were more likely to act on a given opportunity when it appeared in a poor environment compared to a rich one (poor vs. rich: OR = 0.24 [0.19, 0.30], z = 12.56, p < 0.001), and this difference increased at higher expected values (environment × expected value: OR = 0.20 [0.16, 0.24], z = 16.29, p < 0.001).

When we examined reward magnitude and probability separately we again found that participants were more likely to choose to act on opportunities with higher reward magnitudes (OR = 1.20 [1.17, 1.23], z = 15.49, p < 0.001) and with higher reward probabilities (OR = 7.40 [5.52, 10.04], z = 13.27, p < 0.001). Reward magnitude significantly interacted with both recipient (magnitude × recipient: OR = 0.93 [0.91, 0.95], z = 5.73, p < 0.001) and environment (magnitude × environment: OR = 0.89 [0.87, 0.91], z = 9.41, p < 0.001), showing that participants accepted high magnitude offers more often for themselves and more often in the poor environment. We also found that the reward probability interacted with environment (probability × environment: OR = 0.63 [0.44, 0.89], z = 2.64, p = 0.008) wherein participants were more likely to accept higher probability offers in the poor environment relative to the rich one, but we did not see any significant interaction between reward probability and recipient (probability × recipient: OR = 0.94 [0.63, 1.39], z = 0.32, p = 0.75). See **Table S10** for all results from the model.

Finally, as in studies 1 and 2, we found that participants were very successful in the effort task when they chose to act on the opportunity for both self and other (M = 98.7%). We found that participants' effort thresholds measured at the end of the study were lowered compared to those at the beginning ($t_{(53)}$ = 2.45, p = 0.018, d = 0.33 [0.08, 0.57]). We also replicated effects of an interaction between recipient and environment in the amount of credits earned (**Figure S1**). Specifically, participants earned more credits in rich blocks than in poor blocks (poor vs. rich: b = 296.34 [275.71, 316.98], z = 28.59, p < 0.001) and more for themselves that for the

other player (self vs. other: b = –58.66 [–73.60, –43.71], z = 7.74, $p < 0.001$). This self–other difference in credits earned was greater in the rich compared to the poor environment (recipient × environment: b = –74.72 [–104.61, –44.84], z = 4.93, $p < 0.001$). As in the other studies, the majority of participants in study 3 had not seen the movie before (45 *no* vs. 9 *yes*, $\chi^2_{(1)} = 24.00$, $p < 0.001$) and overall enjoyment was high (M (SD) = 7.59 (1.47), 0 = *did not enjoy at all,* 9 = *very much enjoyed*; *t*-test against neutral rating: $t_{(53)} = 15.51$, $p < 0.001$, d = 2.11 [1.59, 3.05]). Altogether, our findings from study 3 demonstrate that the effects of environment and recipient on decisions to act are robust across types of effort and the context in which participants meet another person.

All studies

After completing the main task, we asked participants to rate how they felt when the won or did not win money (0 = *very negative*, 9 = *very positive*) for themselves or for the anonymous other person. Across all studies we found that participants felt more positively about winning money for themselves (M (SD) = 7.67 (1.20), *t*-test against neutral rating: $t(509) = 59.55$, $p <$ 0.001, d = 2.64 [2.41, 2.89] and for others (M (SD) = 6.44 (1.52), $t(509) = 28.83$, $p < 0.001$, d = 1.28 [1.15, 1.41]) compared to when they did not win money (self M (SD) = 3.20 (1.50), $t(509) = 19.45$, $p < 0.001$, d = –0.86 [–0.97, –0.74]; other M (SD) = 3.95 (1.31), $t(509) = 9.49$, $p < 0.001$, d = –0.42 [–0.52, –0.34]). The interaction between recipient and win/not win was significant and showed that participants felt more positive after winning and more negative after not winning money for themselves as compared to how they felt about the outcome when it affected the other person (b = 1.97 [1.73, 2.21], z = 16.35, $p < 0.001$)."

We also asked participants to rate how likely they were to respond to the opportunities in each environment and for each recipient (0 = *much less likely to respond*, 9 = *much more likely to respond*). Participants rated that they were overall more likely to respond in rich environments than in poor environments (b = 3.04 [2.79, 3.29], z = 23.77, $p < 0.001$), and rated they were more likely to respond for themselves than for others (b = 0.83 [0.67, 1.00], z = 10.01, $p <$ 0.001). The interaction between environment and recipient was not significant (b = –0.04 [–0.24, 0.15], z = 0.43, p = 0.67; rich/self: M (SD) = 7.08 (1.61), $t(509) = 36.15$, $p < 0.001$, d = 1.60 [1.46,1.77]; rich/other: M (SD) = 6.29 (1.94), $t(509) = 20.7$, $p < 0.001$, d = 0.92 [0.79, 1.06]; poor/self: M (SD) = 4.08 (1.90), $t(509) = 4.98$, $p < 0.001$, d = –0.22 [–0.32, –0.13]; poor/other: M (SD) = 3.25 (2.01), $t(509) = 14.13$, $p < 0.001$, d = –0.63 [–0.72, –0.53])."

We conducted an exploratory control analysis of our data to test whether the environment*recipient interaction was present when not accounting for expected value using data pooled from the 3 studies to ensure sufficient power. This revealed a significant 2-way interaction between recipient and environment (OR = 0.81 [0.71, 0.92], $z = 3.32$, $p < 0.001$), which provides further support for the differences between poor and rich environments influencing decisions to act to help. However, this analysis assumes that choices are not influenced by low vs. high value rewards, and so we interpret it with some caution. An additional exploratory control analysis examined choices only from trials that were in the central cells of Figure 1c (i.e., those that were medium magnitude and medium probability). Here we found a significant 2-way interaction between recipient and environment (OR = 0.54 [0.35, 0.86], $z = 2.67$, $p = 0.008$) that was also independent of expected value.

Based on the results above, our reported findings do not appear due to simply a framing effect. That is, the difference in choices between poor and rich environments is not driven solely by a difference in context, but also by the value of the current opportunity. In other words, in poor environments a low-value reward is still worth less than a high-value reward, but because high-value rewards are less prevalent, pursuing lower-value opportunities can help to maximise rewards.

Table S1. *GLMM predicting choices (study 1)*

**Fixed effect**	**OR**	**CI low**	**CI high**	***z***	***p***
(Intercept)	3.00	2.41	3.76	9.70	< 0.001
Environment (Poor vs. Rich)	0.50	0.40	0.62	6.36	< 0.001
Recipient (Self vs. Other)	0.14	0.10	0.19	12.94	< 0.001
Expected value	8.93	7.51	10.67	24.50	< 0.001
Environment × Recipient	1.24	0.88	1.77	1.22	0.222
Environment × Expected value	0.32	0.26	0.39	10.44	< 0.001
Recipient × Expected value	0.48	0.39	0.59	6.94	< 0.001
Environment × Recipient × Expected value	1.49	1.05	2.12	2.20	0.028

**Random effect**	***SD***	**Correlations**						
(Intercept)	1.67							
Environment (Poor vs. Rich)	1.37	–0.43						
Recipient (Self vs. Other)	2.12	0.21	–0.30					
Expected value	1.21	0.51	–0.69	0.29				
Environment × Recipient	1.91	0.02	0.09	–0.43	–0.15			
Environment × Expected value	1.13	–0.51	0.76	–0.34	–0.74	0.05		
Recipient × Expected value	1.01	0.38	–0.09	0.62	0.05	–0.24	–0.06	
Environment × Recipient × Expected value	1.19	–0.31	0.12	–0.45	–0.15	0.44	–0.19	–0.81

Note. GLMM: generalised linear mixed-effects model; OR: odds ratio; CI: 95% confidence interval lower/upper bound. All tests were two-sided Wald *Z*-tests.

Table S2. *GLMM with covariates predicting choices (Study 1)*

**Fixed effect**	**OR**	**CI low**	**CI high**	***z***	***p***
(Intercept)	2.92	2.35	3.65	9.55	< 0.001
Trial number	0.52	0.48	0.57	14.95	< 0.001
Previous choice	1.22	1.10	1.36	3.62	< 0.001
Previous expected value	0.97	0.93	1.01	1.66	0.097
Environment (Poor vs. Rich)	0.56	0.46	0.67	6.05	< 0.001
Recipient (Self vs. Other)	0.15	0.11	0.20	12.63	< 0.001
Expected value	9.20	7.74	10.97	25.08	< 0.001
Environment × Recipient	1.00	0.78	1.29	0.02	0.988
Environment × Expected value	0.31	0.25	0.37	11.91	< 0.001
Recipient × Expected value	0.50	0.41	0.60	7.21	< 0.001
Environment × Recipient × Expected value	1.46	1.13	1.88	2.88	0.004

**Random effect**	***SD***	**Correlations**							
(Intercept)	1.65								
Trial number	0.42	0.20							
Previous choice	0.53	0.17	–0.20						
Environment	1.07	–0.47	–0.08	0.00					
Recipient	2.08	0.15	0.16	–0.41	–0.28				
Expected value	1.20	0.49	–0.16	–0.05	–0.65	0.22			
Environment × Recipient	0.89	0.13	0.02	0.32	–0.34	0.10	–0.08		
Environment × Expected value	1.00	–0.51	0.11	0.18	0.74	–0.31	–0.75	–0.08	
Recipient × Expected value	0.84	0.35	0.10	–0.32	–0.13	0.54	–0.01	0.19	–0.18

Note. The maximal model including all random effects failed to converge. Random effects were removed starting with those that contributed the least variance until the model converged. GLMM: generalised linear mixed-effects model; OR: odds ratio; CI: 95% confidence interval lower/upper bound; Previous choice: the accept/reject decision to act on the immediately preceding opportunity; Previous expected value: the expected value of the preceding opportunity. All tests were two-sided Wald *Z*-tests.

Table S3. *GLMM with reward magnitude and probability as separate effects (study 1)*

**Fixed effect**	**OR**	**CI low**	**CI high**	***z***	***p***
(Intercept)	1.91	1.52	2.40	5.51	< 0.001
Magnitude	2.33	2.09	2.60	15.02	< 0.001
Probability	7.05	6.00	8.32	23.51	< 0.001
Recipient (Self vs. Other)	0.13	0.10	0.18	13.21	< 0.001
Environment (Poor vs. Rich)	0.73	0.60	0.89	3.16	0.002
Magnitude × Probability	1.02	0.96	1.09	0.71	0.475
Magnitude × Recipient	0.92	0.82	1.03	1.44	0.149
Probability × Recipient	0.83	0.71	0.97	2.29	0.022
Magnitude × Environment	0.95	0.85	1.06	0.88	0.380
Probability × Environment	1.02	0.87	1.18	0.21	0.837
Recipient × Environment	0.79	0.56	1.13	1.29	0.196
Magnitude × Probability × Recipient	0.89	0.79	1.00	1.95	0.052
Magnitude × Probability × Environment	1.09	0.96	1.23	1.37	0.169
Magnitude × Recipient × Environment	1.08	0.87	1.35	0.72	0.473
Probability × Recipient × Environment	1.13	0.85	1.52	0.85	0.394
Magnitude × Probability × Recipient × Environment	1.17	0.92	1.48	1.29	0.198

**Random effect**	***SD***	**Correlations**							
(Intercept)	1.74								
Magnitude	0.74	−0.15							
Probability	1.16	0.06	0.19						
Recipient	2.18	0.18	0.07	0.30					
Environment	1.22	0.03	−0.40	−0.35	−0.29				
Probability × Recipient	0.69	0.48	0.01	0.24	0.36	−0.02			
Probability × Environment	0.61	−0.20	0.31	0.05	−0.10	−0.22	0.03		
Recipient × Environment	2.07	0.18	−0.22	−0.17	−0.20	0.14	0.19	−0.03	
Probability × Recipient × Environment	1.06	0.06	−0.14	−0.10	0.13	0.21	−0.21	−0.23	−0.14

Note. Magnitude and probability represent the reward's number of credits and probability, respectively. The maximal model including all random effects failed to converge. Random effects were removed starting with those that contributed the least variance until the model converged. GLMM: generalised linear mixed-effects model; OR: odds ratio; CI: 95% confidence interval lower/upper bound. All tests were two-sided Wald *Z*-tests.

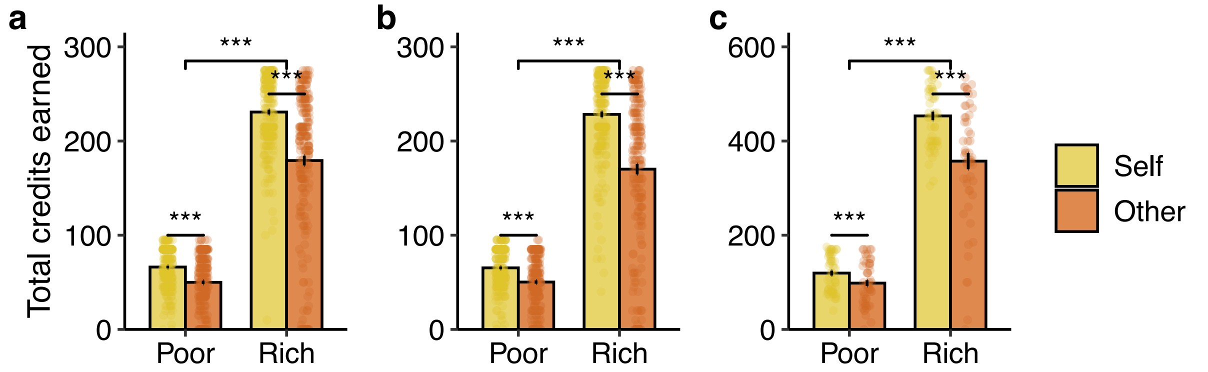

Figure S1. Total number of credits earned within the prosocial ecology task. Mirroring their choice behaviour, participants earned relatively more credits for others in poor environments compared to rich ones than they did for themselves. **(a)** Total number of credits earned in study 1 (n = 237). **(b)** Total number of credits earned in study 2 (n = 219). **(c)** Total number of credits earned in study 3 (n = 54).

Table S4. *GLMM predicting choices (study 2)*

**Fixed effect**	**OR**	**CI low**	**CI high**	***z***	***p***
(Intercept)	3.40	2.68	4.34	9.95	< 0.001
Environment (Poor vs. Rich)	0.29	0.22	0.37	9.59	< 0.001
Recipient (Self vs. Other)	0.10	0.07	0.13	13.52	< 0.001
Expected value	11.60	9.46	14.31	23.34	< 0.001
Environment × Recipient	2.27	1.49	3.54	3.74	< 0.001
Environment × Expected value	0.17	0.13	0.22	14.28	< 0.001
Recipient × Expected value	0.37	0.29	0.46	8.45	< 0.001
Environment × Recipient × Expected value	2.55	1.67	4.01	4.21	< 0.001

**Random effect**	***SD***	**Correlations**						
(Intercept)	1.72							
Environment	1.55	−0.56						
Recipient	2.27	−0.10	0.14					
Expected value	1.38	0.68	−0.72	−0.06				
Environment × Recipient	2.30	0.40	−0.26	−0.48	0.30			
Environment × Expected value	1.16	−0.71	0.81	0.17	−0.86	−0.41		
Recipient × Expected value	1.03	−0.20	0.29	0.64	−0.35	−0.57	0.47	
Environment × Recipient × Expected value	1.76	0.35	−0.48	−0.55	0.46	0.72	−0.62	−0.75

Note. GLMM: generalised linear mixed-effects model; OR: odds ratio; CI: 95% confidence interval lower/upper bound. All tests were two-sided Wald *Z*-tests.

Table S5. *GLMM with covariates predicting choices (study 2)*

**Fixed effect**	**OR**	**CI low**	**CI high**	***z***	***p***
(Intercept)	3.38	2.66	4.33	9.84	< 0.001
Trial number	0.51	0.46	0.55	14.58	< 0.001
Previous choice	1.15	1.03	1.30	2.43	0.015
Previous expected value	0.95	0.91	0.99	2.39	0.017
Environment (Poor vs. Rich)	0.32	0.25	0.40	9.65	< 0.001
Recipient (Self vs. Other)	0.10	0.07	0.14	13.50	< 0.001
Expected value	11.85	9.63	14.68	23.08	< 0.001
Environment × Recipient	1.84	1.26	2.77	3.04	0.002
Environment × Expected value	0.16	0.13	0.20	15.24	< 0.001
Recipient × Expected value	0.37	0.29	0.47	8.18	< 0.001
Environment × Recipient × Expected value	2.51	1.65	3.92	4.18	< 0.001

**Random effect**	***SD***	**Correlations**								
(Intercept)	1.73									
Trial number	0.43	0.39								
Previous choice	0.56	–0.20	–0.29							
Environment	1.23	–0.62	–0.14	0.09						
Recipient	2.19	–0.14	0.05	–0.28	0.19					
Expected value	1.41	0.65	0.12	–0.20	–0.79	–0.07				
Environment × Recipient	1.79	0.34	0.30	0.25	–0.35	–0.49	0.26			
Environment × Expected value	1.10	–0.68	–0.21	0.05	0.85	0.20	–0.87	–0.40		
Recipient × Expected value	1.07	–0.20	–0.15	–0.25	0.47	0.60	–0.38	–0.69	0.53	
Environment × Recipient × Expected value	1.72	0.33	0.22	0.10	–0.55	–0.52	0.45	0.90	–0.57	–0.78

Note. The maximal model including all random effects failed to converge. Random effects were removed starting with those that contributed the least variance until the model converged. GLMM: generalised linear mixed-effects model; OR: odds ratio; CI: 95% confidence interval lower/upper bound; Previous choice: the accept/reject decision to act on the immediately preceding opportunity; Previous expected value: the expected value of the preceding opportunity. All tests were two-sided Wald *Z*-tests.

Table S6. *GLMM predicting choices (study 3)*

**Fixed effect**	**OR**	**CI low**	**CI high**	***z***	***p***
(Intercept)	1.88	1.31	2.71	3.47	< 0.001
Environment (Poor vs. Rich)	0.24	0.19	0.30	12.56	< 0.001
Recipient (Self vs. Other)	0.20	0.13	0.31	7.72	< 0.001
Expected value	18.31	14.15	24.10	21.88	< 0.001
Environment × Recipient	1.75	1.36	2.26	4.31	< 0.001
Environment × Expected value	0.20	0.16	0.24	16.29	< 0.001
Recipient × Expected value	0.32	0.23	0.43	7.32	< 0.001
Environment × Recipient × Expected value	1.92	1.32	2.80	3.40	< 0.001

**Random effect**	***SD***	**Correlations**			
(Intercept)	1.30				
Environment	0.68	−0.44			
Recipient	1.40	−0.14	−0.22		
Expected value	0.87	0.73	−0.34	0.00	
Recipient × Expected value	0.75	−0.26	0.01	0.62	−0.44

Note. The maximal model including all random effects failed to converge. Random effects were removed starting with those that contributed the least variance until the model converged. GLMM: generalised linear mixed-effects model; OR: odds ratio; CI: 95% confidence interval lower/upper bound. All tests were two-sided Wald *Z*-tests.

Table S7. *GLMM with covariates predicting choices (study 3)*

**Fixed effect**	**OR**	**CI low**	**CI high**	***z***	***p***
(Intercept)	2.10	1.37	3.25	3.44	< 0.001
Trial number	0.65	0.59	0.71	8.93	< 0.001
Previous choice	0.92	0.78	1.10	0.88	0.379
Previous expected value	0.86	0.81	0.92	4.49	< 0.001
Environment (Poor vs. Rich)	0.19	0.14	0.26	10.35	< 0.001
Recipient (Self vs. Other)	0.20	0.13	0.30	7.77	< 0.001
Expected value	22.65	16.40	31.84	18.82	< 0.001
Environment × Recipient	1.76	1.35	2.29	4.18	< 0.001
Environment × Expected value	0.12	0.08	0.17	11.17	< 0.001
Recipient × Expected value	0.33	0.24	0.44	7.41	< 0.001
Environment × Recipient × Expected value	2.05	1.40	3.01	3.68	< 0.001

**Random effect**	***SD***	**Correlations**						
(Intercept)	1.55							
Trial number	0.30	0.27						
Previous choice	0.46	–0.09	–0.07					
Environment	0.97	–0.78	–0.18	0.25				
Recipient	1.40	–0.03	0.13	–0.39	–0.21			
Expected value	1.12	0.81	0.22	–0.07	–0.83	0.08		
Environment × Expected value	1.07	–0.84	–0.36	0.23	0.96	–0.21	–0.92	
Recipient × Expected value	0.68	–0.07	0.16	0.00	0.02	0.71	–0.24	0.05

Note. The maximal model including all random effects failed to converge. Random effects were removed starting with those that contributed the least variance until the model converged. GLMM: generalised linear mixed-effects model; OR: odds ratio; CI: 95% confidence interval lower/upper bound; Previous choice: the accept/reject decision to act on the immediately preceding opportunity; Previous expected value: the expected value of the preceding opportunity. All tests were two-sided Wald *Z*-tests.

Table S8. *Fits of computational models on study 1*

**Model parameters**		**Model fits**		
**Opportunity cost**	**Value sensitivity**	**XP**	**intBIC**	**R^2**
1o	1β	0.000	32000	0.372
1o	2$\beta_{self/other}$	0.000	30212	0.427
1o	2$\beta_{poor/rich}$	0.000	31857	0.388
1o	4β	0.000	30295	0.454
2$o_{self/other}$	1β	0.002	27677	0.485
2$o_{self/other}$	2$\beta_{self/other}$	0.003	27538	0.501
2$o_{self/other}$	2$\beta_{poor/rich}$	0.000	27603	0.499
2$o_{self/other}$	4β	0.000	27511	0.521
2$o_{poor/rich}$	1β	0.000	31880	0.391
2$o_{poor/rich}$	2$\beta_{self/other}$	0.000	30388	0.445
2$o_{poor/rich}$	2$\beta_{poor/rich}$	0.000	31961	0.401
2$o_{poor/rich}$	4β	0.000	30544	0.468
4o	1β	**0.994**	**27428**	0.530
4o	2$\beta_{self/other}$	0.000	27472	0.539
4o	2$\beta_{poor/rich}$	0.000	27682	0.538
4o	4β	0.001	27718	**0.550**

Note: Presented here are the model fits for the models that included a single risk aversion (α) parameter. Bolded values are the best fitting models from the given column. XP = exceedance probability, intBIC = integrated Bayesian Information Criterion.

Table S9. *Fits of computational models on study 2*

**Model parameters**		**Model fits**		
**Opportunity cost**	**Value sensitivity**	**XP**	**intBIC**	**R^2**
$1o$	1β	0.000	30527	0.349
$1o$	$2\beta_{self/other}$	0.000	28798	0.408
$1o$	$2\beta_{poor/rich}$	0.000	30283	0.368
$1o$	4β	0.000	28789	0.436
$2o_{self/other}$	1β	0.000	26015	0.474
$2o_{self/other}$	$2\beta_{self/other}$	0.000	25899	0.489
$2o_{self/other}$	$2\beta_{poor/rich}$	0.000	25864	0.494
$2o_{self/other}$	4β	0.000	25687	0.516
$2o_{poor/rich}$	1β	0.000	30331	0.370
$2o_{poor/rich}$	$2\beta_{self/other}$	0.000	28881	0.429
$2o_{poor/rich}$	$2\beta_{poor/rich}$	0.000	30316	0.380
$2o_{poor/rich}$	4β	0.000	28918	0.452
$4o$	1β	0.006	25640	0.521
$4o$	$2\beta_{self/other}$	0.000	**25537**	0.533
$4o$	$2\beta_{poor/rich}$	0.000	25678	0.530
$4o$	4β	**0.994**	25749	**0.544**

Note: Presented here are the model fits for the models that included a single risk aversion (α) parameter. Bolded values are the best fitting models from the given column. XP = exceedance probability, intBIC = integrated Bayesian Information Criterion.

Table S10. *Fits of computational models on study 3*

**Model parameters**		**Model fits**		
**Opportunity cost**	**Value sensitivity**	**XP**	**intBIC**	**R^2**
1o	1β	0.000	12770	0.438
1o	2$\beta_{self/other}$	0.000	12534	0.461
1o	2$\beta_{poor/rich}$	0.000	12713	0.448
1o	4β	0.001	12456	0.479
2$o_{self/other}$	1β	0.001	11527	0.509
2$o_{self/other}$	2$\beta_{self/other}$	0.002	11470	0.517
2$o_{self/other}$	2$\beta_{poor/rich}$	0.001	11512	0.518
2$o_{self/other}$	4β	0.002	11507	0.528
2$o_{poor/rich}$	1β	0.029	12357	0.469
2$o_{poor/rich}$	2$\beta_{self/other}$	0.002	12174	0.492
2$o_{poor/rich}$	2$\beta_{poor/rich}$	0.155	12383	0.476
2$o_{poor/rich}$	4β	0.001	12214	0.503
4o	1β	0.001	**11242**	0.546
4o	2$\beta_{self/other}$	0.111	11248	0.555
4o	2$\beta_{poor/rich}$	0.004	11244	0.552
4o	4β	0.030	11295	**0.562**
4o, 2$\beta_{self/other}$, 1α, 1γ		**0.555**	11269	0.556

Note: Presented here are the model fits for the models that included a single risk aversion (α) parameter. Also included is the model with the highest exceedance probability which included a weighting parameter (γ) on the reward probability in addition to the risk aversion parameter. Bolded values are the best fitting models from the given column. XP = exceedance probability, intBIC = integrated Bayesian Information Criterion.

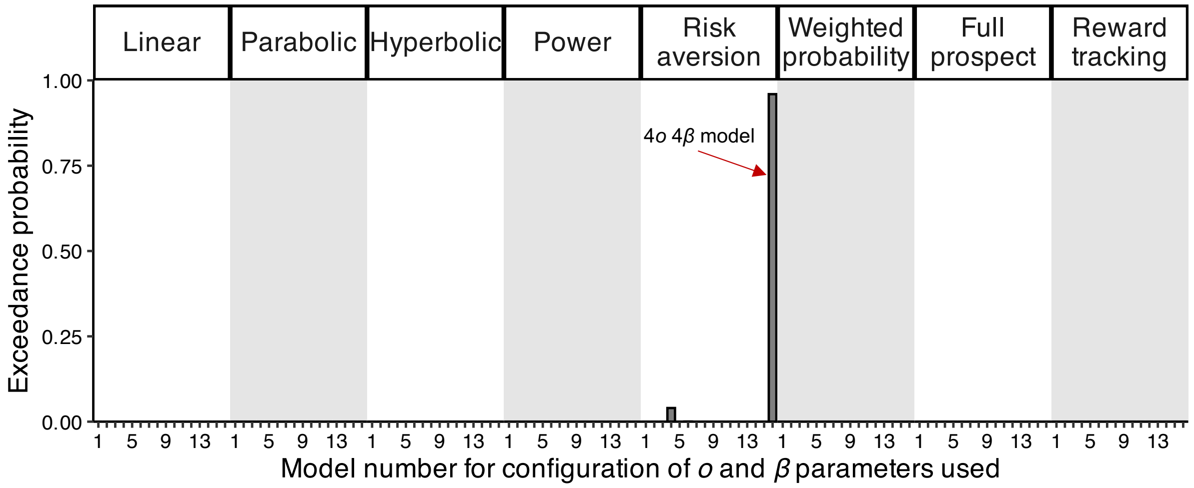

Figure S2. Exceedance probabilities for all 128 model combinations tested. Choice data from the 3 studies were combined to maximise power (n = 510). The model which included 4 separate parameters for opportunity costs and for value sensitivity, in addition to a risk aversion parameter, had the highest exceedance probability (0.96). The model numbers 1-16 on the x-axis represent the following parameter combinations: **(1)** 1o 1β, **(2)** 2$o_{s/o}$ 1β, **(3)** 2$o_{p/r}$ 1β, **(4)** 4o 1β, **(5)** 1o 2$\beta_{s/o}$, **(6)** 2$o_{s/o}$ 2$\beta_{s/o}$, **(7)** 2$o_{p/r}$ 2$\beta_{s/o}$, **(8)** 4o 2$\beta_{s/o}$, **(9)** 1o 2$\beta_{p/r}$, **(10)** 2$o_{s/o}$ 2$\beta_{p/r}$, **(11)** 2$o_{p/r}$ 2$\beta_{p/r}$, **(12)** 4o 2$\beta_{p/r}$, **(13)** 1o 4β, **(14)** 2$o_{s/o}$ 4β, **(15)** 2$o_{p/r}$ 4β, **(16)** 4o 4β. The o and β parameters represent the opportunity cost and value sensitivity parameters, respectively. The s, o, p, and r subscripts represent the self, other, poor, and rich conditions, respectively.

Table S11. *Correlations between the factors and model parameters*

	Psychiatric traits	Empathy and Emotional Motivation	Utilitarianism
$o_{self/poor}$	$r_{(506)} = 0.02$, $p = 0.680$<br>$BF_{01} = 15.84$	$r_{(506)} = –0.03$, $p = 0.585$<br>$BF_{01} = 13.86$	$r_{(506)} = –0.06$, $p = 0.293$<br>$BF_{01} = 8.05$
$o_{self/rich}$	$r_{(506)} = 0.04$, $p = 0.494$<br>$BF_{01} = 12.14$	$r_{(506)} = –0.01$, $p = 0.859$<br>$BF_{01} = 17.66$	$r_{(506)} = –0.01$, $p = 0.847$<br>$BF_{01} = 17.54$
$o_{other/poor}$	$r_{(506)} = 0.02$, $p = 0.669$<br>$BF_{01} = 15.45$	$r_{(506)} = –0.10$, $p = 0.046$<br>$BF_{01} = 1.11$	$r_{(506)} = –0.14$, $p = 0.007$<br>$BF_{01} = 0.17$
$o_{other/rich}$	$r_{(506)} = 0.08$, $p = 0.134$<br>$BF_{01} = 3.70$	$r_{(506)} = –0.11$, $p = 0.034$<br>$BF_{01} = 0.78$	$r_{(506)} = –0.18$, $p < 0.001$<br>$BF_{01} = 0.00$
$\beta_{self/poor}$	$r_{(506)} = –0.03$, $p = 0.634$<br>$BF_{01} = 14.89$	$r_{(506)} = 0.06$, $p = 0.242$<br>$BF_{01} = 6.88$	$r_{(506)} = –0.08$, $p = 0.105$<br>$BF_{01} = 2.91$
$\beta_{self/rich}$	$r_{(506)} = –0.04$, $p = 0.453$<br>$BF_{01} = 11.20$	$r_{(506)} = 0.06$, $p = 0.226$<br>$BF_{01} = 6.31$	$r_{(506)} = –0.09$, $p = 0.097$<br>$BF_{01} = 2.58$
$\beta_{other/poor}$	$r_{(506)} = 0.03$, $p = 0.619$<br>$BF_{01} = 14.58$	$r_{(506)} = 0.03$, $p = 0.585$<br>$BF_{01} = 13.86$	$r_{(506)} = –0.08$, $p = 0.152$<br>$BF_{01} = 4.35$
$\beta_{other/rich}$	$r_{(506)} = –0.07$, $p = 0.152$<br>$BF_{01} = 4.37$	$r_{(506)} = –0.02$, $p = 0.694$<br>$BF_{01} = 16.15$	$r_{(506)} = –0.10$, $p = 0.069$<br>$BF_{01} = 1.79$
α	$r_{(506)} = 0.04$, $p = 0.490$<br>$BF_{01} = 11.92$	$r_{(506)} = –0.09$, $p = 0.091$<br>$BF_{01} = 2.38$	$r_{(506)} = –0.09$, $p = 0.105$<br>$BF_{01} = 2.86$

Note. *r* values represent Pearson's correlation coefficient. Tests were two-sided and FDR-adjusted. BF_{01} represents the Bayes factor in favour of the null (i.e., no correlation between the variables).

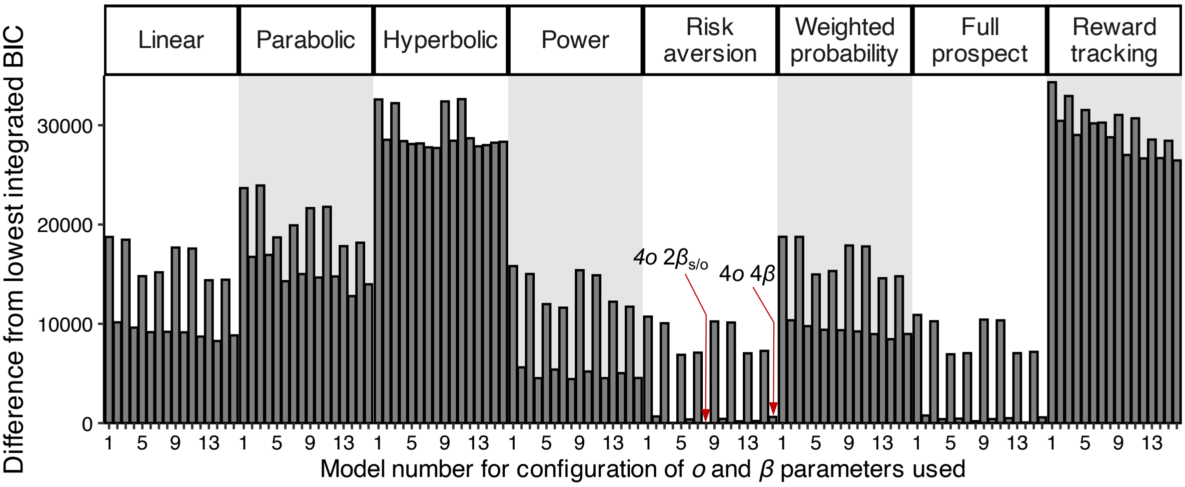

Figure S3. Summed integrated BIC scores for each model. Represented here are the differences in summed integrated BIC (BICint) scores from the model with the lowest summed BICint. Choice data from the 3 studies were combined to maximise power (*n* = 510). The model with four opportunity cost and two value sensitivity parameters (for each recipient), as well as a risk aversion parameter had the lowest summed BICint. The model numbers 1-16 on the x-axis represent the following parameter combinations: **(1)** 1o 1β, **(2)** 2$o_{s/o}$ 1β, **(3)** 2$o_{p/r}$ 1β, **(4)** 4o 1β, **(5)** 1o 2$\beta_{s/o}$, **(6)** 2$o_{s/o}$ 2$\beta_{s/o}$, **(7)** 2$o_{p/r}$ 2$\beta_{s/o}$, **(8)** 4o 2$\beta_{s/o}$, **(9)** 1o 2$\beta_{p/r}$, **(10)** 2$o_{s/o}$ 2$\beta_{p/r}$, **(11)** 2$o_{p/r}$ 2$\beta_{p/r}$, **(12)** 4o 2$\beta_{p/r}$, **(13)** 1o 4β, **(14)** 2$o_{s/o}$ 4β, **(15)** 2$o_{p/r}$ 4β, **(16)** 4o 4β. The o and β parameters represent the opportunity cost and value sensitivity parameters, respectively. The s, o, p, and r subscript represent the self, other, poor, and rich conditions, respectively.

Table S12. *GLMM with reward magnitude and probability as separate effects (study 2)*

**Fixed effect**	**OR**	**CI low**	**CI high**	***z***	***p***
(Intercept)	1.89	1.17	1.23	5.38	< 0.001
Magnitude	2.31	5.52	10.04	13.88	< 0.001
Probability	7.89	0.25	0.74	27.44	< 0.001
Recipient	0.11	0.67	1.44	13.38	< 0.001
Environment	0.62	1.06	1.11	4.80	< 0.001
Magnitude × Probability	1.06	0.91	0.95	1.40	0.163
Magnitude × Recipient	0.98	0.63	1.39	0.38	0.702
Probability × Recipient	0.74	0.87	0.91	3.83	< 0.001
Magnitude × Environment	0.78	0.44	0.89	3.71	< 0.001
Probability × Environment	0.87	0.40	1.24	2.17	0.030
Recipient × Environment	0.80	0.93	0.98	1.30	0.192
Magnitude × Probability × Recipient	1.19	0.97	1.03	2.66	0.008
Magnitude × Probability × Environment	1.14	1.00	1.10	1.73	0.083
Magnitude × Recipient × Environment	0.79	0.47	1.71	1.91	0.056
Probability × Recipient × Environment	0.72	0.97	1.09	2.43	0.015
Magnitude × Probability × Recipient × Environment	0.74	1.17	1.23	2.31	0.021

**Random effect**	***SD***	**Correlations***
(Intercept)	1.69	
Magnitude	0.77	
Probability	0.98	
Recipient	2.30	
Environment	1.24	
Magnitude × Probability	0.30	
Probability × Recipient	0.70	
Magnitude × Environment	0.45	
Probability × Environment	0.48	
Recipient × Environment	2.06	
Magnitude × Probability × Environment	0.57	
Magnitude × Recipient × Environment	0.53	
Probability × Recipient × Environment	1.10	

Note. Magnitude and probability represent the reward's number of credits and probability, respectively. The maximal model including all random effects failed to converge. Random effects were removed starting with those that contributed the least variance until the model converged. *The best fitting model removed covariances between the random effects. GLMM: generalised linear mixed-effects model; OR: odds ratio; CI: 95% confidence interval lower/upper bound.

Study 3

Table S13. *GLMM with reward magnitude and probability as separate effects (study 3)*

Fixed effect	OR	CI low	CI high	*z*	*p*
(Intercept)	0.12	0.08	0.19	9.17	< 0.001
Magnitude	1.20	1.17	1.23	15.49	< 0.001
Probability	7.40	5.52	10.04	13.27	< 0.001
Recipient	0.44	0.25	0.74	3.09	0.002
Environment	0.98	0.67	1.44	0.09	0.926
Magnitude × Probability	1.09	1.06	1.11	8.31	< 0.001
Magnitude × Recipient	0.93	0.91	0.95	5.73	< 0.001
Probability × Recipient	0.94	0.63	1.39	0.32	0.745
Magnitude × Environment	0.89	0.87	0.91	9.41	< 0.001
Probability × Environment	0.63	0.44	0.89	2.64	0.008
Recipient × Environment	0.71	0.40	1.24	1.20	0.231
Magnitude × Probability × Recipient	0.95	0.93	0.98	3.10	0.002
Magnitude × Probability × Environment	1.00	0.97	1.03	0.19	0.849
Magnitude × Recipient × Environment	1.05	1.00	1.10	1.90	0.058
Probability × Recipient × Environment	0.90	0.47	1.71	0.33	0.745
Magnitude × Probability × Recipient × Environment	1.03	0.97	1.09	0.90	0.371

Random effect	*SD*	Correlations*
(Intercept)	1.58	
Magnitude	0.07	
Probability	0.85	
Recipient	1.63	
Environment	0.94	
Magnitude × Probability	0.04	
Probability × Recipient	0.78	
Probability × Environment	0.39	
Magnitude × Probability × Environment	0.04	
Probability × Recipient × Environment	0.27	

Note. Magnitude and probability represent the reward's number of credits and probability, respectively. The maximal model including all random effects failed to converge. Random effects were removed starting with those that contributed the least variance until the model converged. *The best fitting model removed covariances between the random effects. GLMM: generalised linear mixed-effects model; OR: odds ratio; CI: 95% confidence interval lower/upper bound.

Instructions for online version of Prosocial Ecology Task

The instructions for the in-person version (study 3) were identical except for the description of the effort task and the other player (whom they met in person). The Prolific ID shown was randomised for each online participant (study 1 and study 2).

In this task you will watch a movie. While watching the movie you will have opportunities to briefly stop the video and click the mouse for potential rewards. These rewards may be given to either you or another online player on Prolific who is completing a different task. This user’s Prolific ID is 5b3c3474cde973bd647e933

You have been assigned the role of *Player 1* and user 5b3c3474cde973bd647e933 has been assigned the role of *Player 2*. This means you will be making decisions that impact Player 2, but they will not be making decisions that impact you.

You will have opportunities to briefly stop the video and click the mouse button in return for potential rewards. Each time an opportunity appears, you will be able to decide whether you want to accept it or continue watching the movie.

When an opportunity appears, you will see an image like the one to the right (coloured ovals with dots inside them).

Each opportunity will be different, and some will be better than others.

The *size* of the reward (i.e., how many credits the opportunity is worth) varies from offer to offer.

The *probability* of the reward being given to you (i.e., the chances that you will actually be paid those credits) also varies from offer to offer.

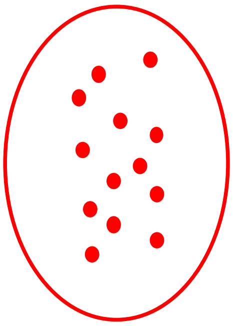

276

The colour of the stimulus indicates the amount of reward that an opportunity is worth.

Red offers are worth 5 credits.

Yellow offers are worth 10 credits.

Purple offers are worth 20 credits.

Credits earned during the task will be converted into £ at the end of the session.

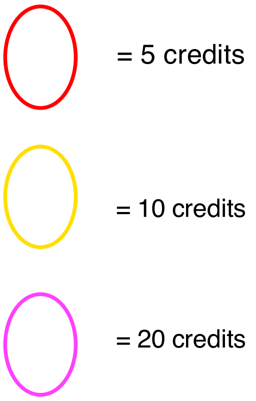

277

The number of dots inside the oval indicates the probability that you will be given the credits. More dots means that you are more likely to gain the credits for that opportunity.

For example, if the oval is nearly full of dots, there is a high probability that the reward will be given. If there are only a few dots in the oval, then reward is unlikely to be given.

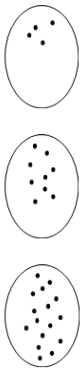

= low probability

= medium probability

= high probability

278

While you are watching the movie, opportunities will appear on screen. If you wish to accept an opportunity, press the SPACEBAR while the coloured oval is on the screen.

The offers appear for a brief amount of time, so don't dwell too long on your decision!

279

If you accept an opportunity, you will need to take a brief break from the movie and *click the mouse button* as fast as you can to be eligible for the reward it (might) provide.

Remember that the height of the red bar indicates how many clicks you have made. Click as fast as you can to get the red bar to reach the green zone at the top.

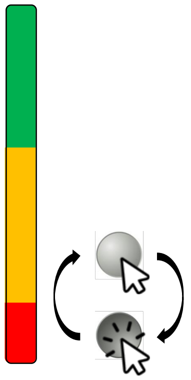

280

If you fail to reach the green zone in time, you will forfeit any possible rewards from that opportunity.

Remember: reaching the green zone does not *guarantee* that you will get a reward – that depends on the reward-probability of the opportunity (i.e., the number of dots in the oval)!

281

You will always be provided with on-screen feedback about whether you earned a reward from an opportunity, regardless of whether or not you responded to it.

Let's walk through an example of what we've discussed so far.

282

Imagine you're watching a movie about the coral reefs of Australia

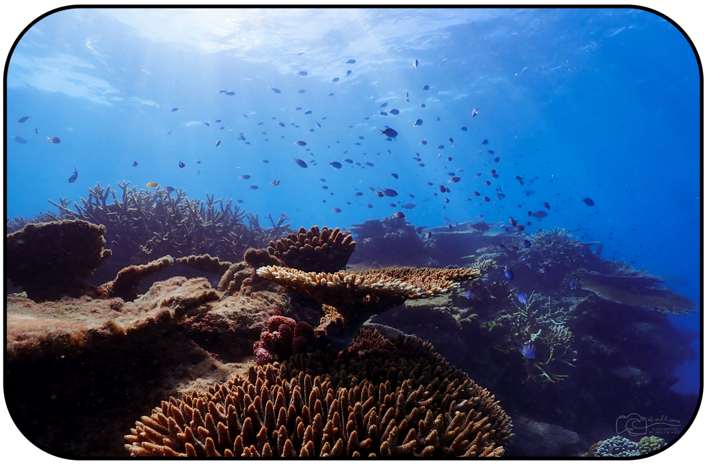

283

…and you see an opportunity appear on screen

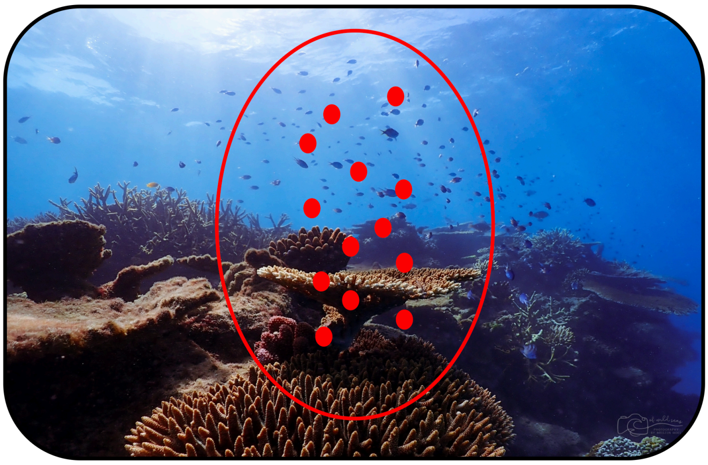

284

You can accept the opportunity by pressing the SPACEBAR (try it now)

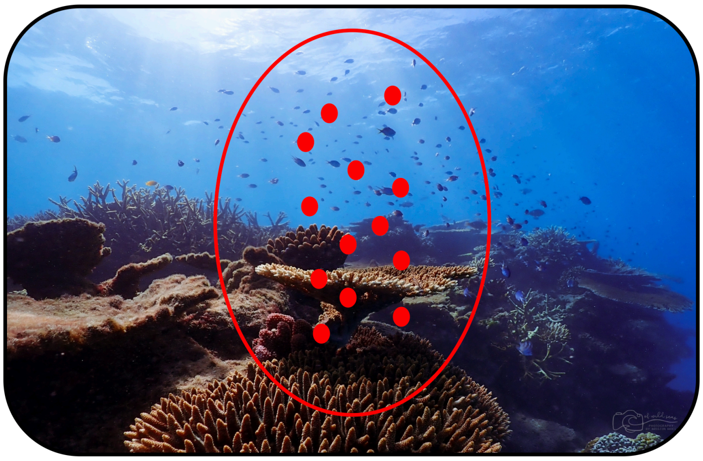

285

...which will transfer you to a screen like this; **click as fast as you can**!

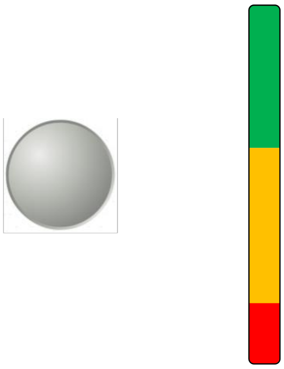

286

The movie will then reappear, and you will get feedback about the outcome of your decision – i.e. whether you earned any credits

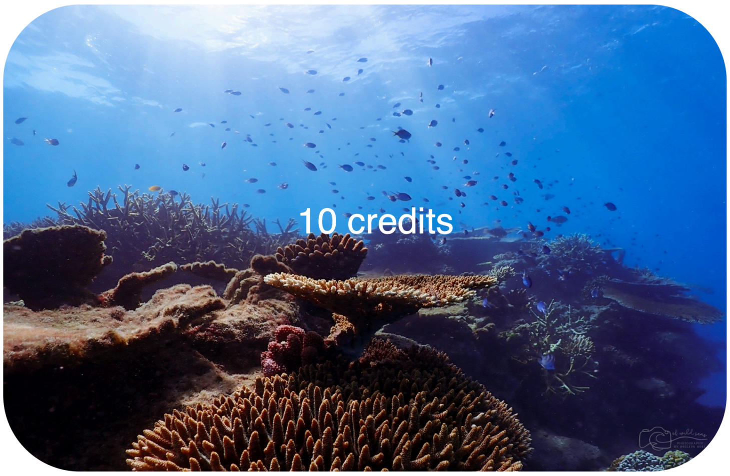

287

The task is divided into four ‘blocks’ of opportunities.

In RICH blocks, the frequency of opportunities worth lots of credits and with high probabilities of giving rewards (i.e., more dots) will be higher.

In POOR blocks, the reverse is true. There will be fewer opportunities worth lots of credits and fewer with high probabilities of giving rewards.

Each time a new block begins, text will appear on screen to tell you what kind of block it is (i.e., rich vs poor).

288

There is another important difference in the blocks: ***who*** you are playing for.

Some blocks will be performed for *yourself*. This means that you are the beneficiary of any credits earned.

Some blocks will be performed for *Player 2*. This means that user 5b3c3474cde973bd647e933 receives the credits accumulated during that block.

Player 2 does not know that you will – on some occasions – be playing on their behalf. Player 2 will perform a similar task, but their decisions will not impact on you.

289

You will be told who you are playing for at the beginning of each block. This information will also be shown above each opportunity as it appears, and when you receive feedback about the outcome(s) of those opportunities.

It is important that you pay attention throughout the task. Occasionally we will check that you are paying attention by asking you to quickly press a button that appears on screen.

You will perform four blocks in total; two for yourself and two for Player 2. For example...

When you are playing for yourself, the opportunities will look like this:

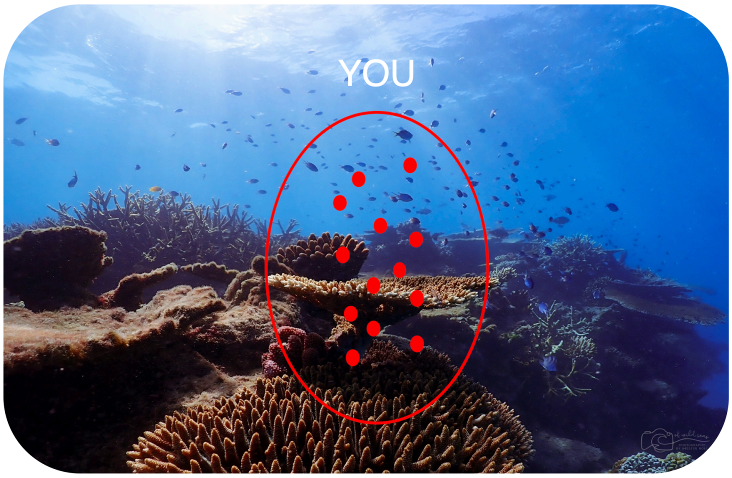

When you are playing for Player 2, the opportunities will look like this:

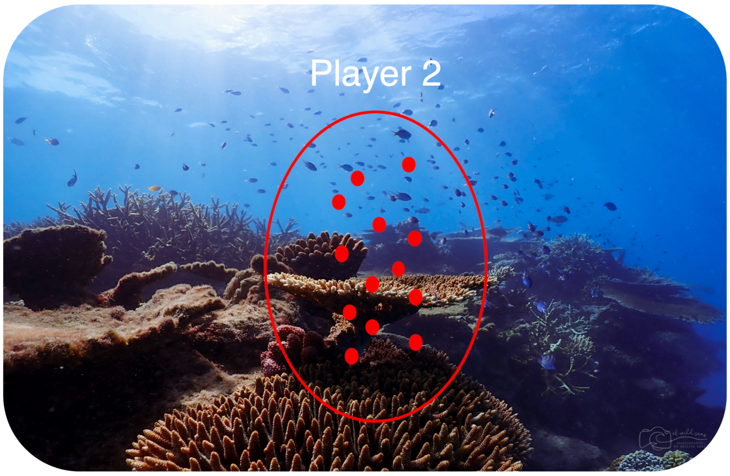

When a button appears on screen, be sure to click it as quickly as possible!

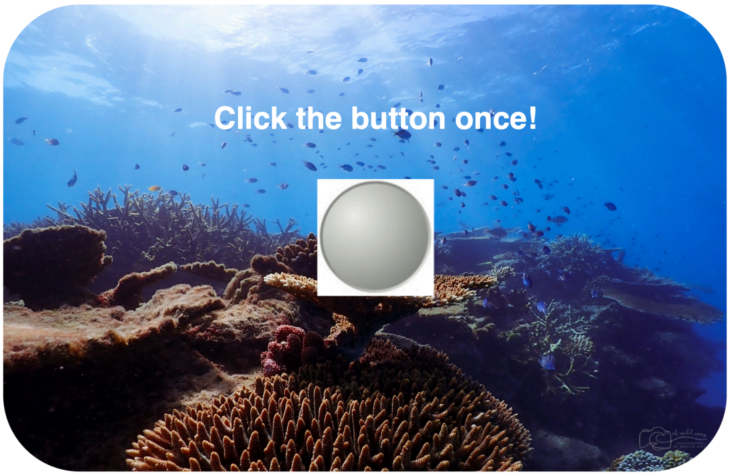

293

Your decisions with respect to opportunities are up to you and will remain completely confidential. Although these decisions are recorded by the computer, this data will not be linked to your name and the experimenters will be unable to identify your choices.

Player 2 will never learn your identity, and you will never learn Player 2's identity. This strict confidentiality was approved by the University of Birmingham's Research Governance, Ethics and Integrity Committee and applies to all the tasks you complete today.

294

Before we begin, let’s do some practice!

Remember, when an opportunity (coloured oval) appears on screen, and you wish to accept it, press the SPACEBAR.

References

1. M. Seedorff, J. Oleson, B. McMurray, “Maybe maximal: Good enough mixed models optimize power while controlling Type I error” (preprint, PsyArXiv, 2019); https://doi.org/10.31234/osf.io/xmhfr.

2. H. Matuschek, R. Kliegl, S. Vasishth, H. Baayen, D. Bates, Balancing Type I error and power in linear mixed models. *J. Mem. Lang.* **94**, 305–315 (2017).
