# Supplementary material for: Humans are more prosocial in poor foraging environments
Source: Nat Commun. 2026 Feb 9;17:483. doi: 10.1038/s41467-025-66880-9 (PMC12887026; doi:10.1038/s41467-025-66880-9)
Supplement: Supplementary file 2 — Reporting Summary [file 41467_2025_66880_MOESM2_ESM.pdf]

nature portfolio

Corresponding author(s): Todd A. Vogel

Last updated by author(s): Nov 5, 2025

Reporting Summary

Nature Portfolio wishes to improve the reproducibility of the work that we publish. This form provides structure for consistency and transparency in reporting. For further information on Nature Portfolio policies, see our Editorial Policies and the Editorial Policy Checklist.

Statistics

For all statistical analyses, confirm that the following items are present in the figure legend, table legend, main text, or Methods section.

n/a	Confirmed	
☐	☒	The exact sample size (n) for each experimental group/condition, given as a discrete number and unit of measurement
☐	☒	A statement on whether measurements were taken from distinct samples or whether the same sample was measured repeatedly
☐	☒	The statistical test(s) used AND whether they are one- or two-sided<br>*Only common tests should be described solely by name; describe more complex techniques in the Methods section.*
☐	☒	A description of all covariates tested
☐	☒	A description of any assumptions or corrections, such as tests of normality and adjustment for multiple comparisons
☐	☒	A full description of the statistical parameters including central tendency (e.g. means) or other basic estimates (e.g. regression coefficient) AND variation (e.g. standard deviation) or associated estimates of uncertainty (e.g. confidence intervals)
☐	☒	For null hypothesis testing, the test statistic (e.g. F, t, r) with confidence intervals, effect sizes, degrees of freedom and P value noted<br>*Give P values as exact values whenever suitable.*
☐	☒	For Bayesian analysis, information on the choice of priors and Markov chain Monte Carlo settings
☐	☒	For hierarchical and complex designs, identification of the appropriate level for tests and full reporting of outcomes
☐	☒	Estimates of effect sizes (e.g. Cohen's d, Pearson's r), indicating how they were calculated

Our web collection on statistics for biologists contains articles on many of the points above.

Software and code

Policy information about availability of computer code

Data collection	Data in studies 1 and 2 were collected using Gorilla Experiment Builder (www.gorilla.sc). Data in study 3 were collected using the Psychophysics Toolbox (version 3.0.11) for MATLAB (version 2012b). Questionnaire data for all studies were collected using Qualtrics survey software (Provo, Utah).
Data analysis	Behavioural data and computational parameters were analysed in R (v4.3.1) using the glmmTMB package (v1.17). Computational modelling was conducted in MATLAB (v2022a) using custom code. Analysis scripts and raw data can be found on the Open Science Framework (https://doi.org/10.17605/osf.io/dmfhq).

For manuscripts utilizing custom algorithms or software that are central to the research but not yet described in published literature, software must be made available to editors and reviewers. We strongly encourage code deposition in a community repository (e.g. GitHub). See the Nature Portfolio guidelines for submitting code & software for further information.

Data

Policy information about availability of data

All manuscripts must include a data availability statement. This statement should provide the following information, where applicable:

- Accession codes, unique identifiers, or web links for publicly available datasets
- A description of any restrictions on data availability
- For clinical datasets or third party data, please ensure that the statement adheres to our policy

The raw datasets analysed during the current study are available at: https://doi.org/10.17605/osf.io/dmfhq

Research involving human participants, their data, or biological material

Policy information about studies with human participants or human data. See also policy information about sex, gender (identity/presentation), and sexual orientation and race, ethnicity and racism.

Reporting on sex and gender	Self-reported gender was used to obtain balanced samples in studies 1 and 2. For all studies, we report the number of participants identifying as man, woman, or other.
Reporting on race, ethnicity, or other socially relevant groupings	Data on race, ethnicity, or other socially relevant groupings were not used in this study.
Population characteristics	See below.
Recruitment	See below.
Ethics oversight	Studies 1 and 2: University of Birmingham Research Ethics Committee; Study 3: University of Birmingham and University of Oxford Research Ethics Committees

Note that full information on the approval of the study protocol must also be provided in the manuscript.

Field-specific reporting

Please select the one below that is the best fit for your research. If you are not sure, read the appropriate sections before making your selection.

☐ Life sciences ☒ Behavioural & social sciences ☐ Ecological, evolutionary & environmental sciences

For a reference copy of the document with all sections, see nature.com/documents/nr-reporting-summary-flat.pdf

Behavioural & social sciences study design

All studies must disclose on these points even when the disclosure is negative.

Study description	Quantitative experimental studies examining choices in a decision-making task with self-report measures of behaviour and traits.
Research sample	Study 1: 237 people (aged 18–35 years, M (SD) = 28.8 (4.4); self-identified gender: 124 women, 112 men, 1 non-binary) recruited through Prolific as a balanced sample from the United Kingdom.<br>Study 2: 219 people (aged 18–35 years, M (SD) = 27.7 (4.8), self-identified gender: 108 women, 107 men, 4 non-binary) recruited through Prolific as a balanced sample from the United Kingdom.<br>Study 3: 54 people (aged 18–32 years, M (SD) = 21.9 (3.2), self-identified gender: 39 women, 15 men) recruited through the University of Birmingham and University of Oxford communities.
Sampling strategy	Online participants were recruited through Prolific to provide a gender-balanced sample from the United Kingdom. In-person participants were recruited through the University of Birmingham and University of Oxford communities using convenience sampling. Sample sizes were determined based on previous studies using a similar paradigm (Khalighinejad et al., Nat Commun, 2021).
Data collection	Participants in studies 1 and 2 completed the study online. Participants in study 3 completed the study on a laptop with a researcher present in the room to provide guidance if necessary.
Timing	Study 1: June 2022 - August 2022<br>Study 2: September 2022 - October 2022<br>Study 3: November 2019 - March 2020 and December 2022 - January 2023
Data exclusions	Participants were excluded from analysis based on preregistered criteria included failing at least 50% of attention checks, overall acceptance rates below 10% or above 90%, as well as unregistered criteria of failing an attention check question in the QCAE, failing to correctly self-identify as Player 1 in debriefing questions. In total, 86 people were excluded in study 1, 82 people in study 2, and 1 person in study 3.

Non-participation	No participants declined participation or withdrew from the compiled dataset.
Randomization	The study used a within-subject design that randomized the task order between participants; no random group allocation was necessary.

Reporting for specific materials, systems and methods

We require information from authors about some types of materials, experimental systems and methods used in many studies. Here, indicate whether each material, system or method listed is relevant to your study. If you are not sure if a list item applies to your research, read the appropriate section before selecting a response.

Materials & experimental systems

n/a	Involved in the study
☒	☐ Antibodies
☒	☐ Eukaryotic cell lines
☒	☐ Palaeontology and archaeology
☒	☐ Animals and other organisms
☒	☐ Clinical data
☒	☐ Dual use research of concern
☒	☐ Plants

Methods

n/a	Involved in the study
☒	☐ ChIP-seq
☒	☐ Flow cytometry
☒	☐ MRI-based neuroimaging

Plants

Seed stocks	NA
Novel plant genotypes	NA
Authentication	NA
